# Community-engaged training in informed consent

**DOI:** 10.1017/cts.2023.534

**Published:** 2023-04-20

**Authors:** Kris M. Markman, Noelle P. Weicker, Andreas K. Klein, Robert Sege

**Affiliations:** 1 Tufts Clinical and Translational Science Institute, Tufts University, Boston, MA, USA; 2 Tufts University School of Medicine, Boston, MA, USA; 3 Institute for Clinical Research and Health Policy Studies, Tufts Medical Center, Boston, MA, USA; 4 Division of Hematology and Oncology, Tufts Medical Center, Boston, MA, USA

**Keywords:** Workforce development, training, community engagement, informed consent, research coordinators

## Abstract

Inadequate training in the interpersonal skills of conducting informed consent conversations has long been noted as a challenge for clinical research recruitment and retention. To address this critical gap, Tufts Clinical and Translational Science Institute developed regular trainings for clinical research coordinators and other research staff on the practical skills of communicating informed consent using community members as simulated patients for role-playing exercises. In this paper, we assess the reach and effectiveness of these trainings and describe the impact of employing community stakeholders as simulated patients. We found that by embedding community members in the trainings, clinical research coordinators get to hear diverse perspectives, experience a range of patient responses, and learn from the lived experience of the communities that research tries to serve. Utilizing community members as trainers also helps to dismantle traditional power dynamics by demonstrating the organization’s commitment to inclusiveness and community engagement. Based on these findings, we suggest that training on informed consent include more simulated consent exercises that feature interaction with community members who can provide real-time feedback to coordinators.

## Introduction

Informed consent is the cornerstone of the human subjects research process. The right to decide for oneself whether or not to participate in research is fundamental and inviolable [1]. Investigators and research team members participate in regular training in the ethics of human subjects research, including informed consent, as a precondition to undertaking research. This training typically focuses on the historical antecedents of contemporary research regulations and the principles which underly them [2]. In addition, most Institutional Review Boards (IRBs) have templates and guidelines for written informed consent documents. However, informed consent is more than the document; it is a process, and a critical part of that process is the conversation [3]. However, inconsistent or sometimes nonexistent training in the practical interpersonal skills of conducting informed consent conversations has long been noted as a challenge for clinical research [4–8].

Experiential learning and hands-on, practical experience in Good Clinical Practice (GCP) is highly valued by research coordinators, and years of experience as a research coordinator is a better predictor of GCP knowledge than hours of training [9]. Educational techniques such as simulations and role-plays can help bridge that gap by including experiential components in a more formalized training setting [10,11]. For example, simulation is effective at teaching a variety of skills in medical education [12] and nursing education [11]. Education that incorporates role-playing – with immediate feedback – improves medical students’ clinical [13,14] and communication [15] skills. Training that incorporates simulated or standardized patients (SPs) is an effective way to provide continuing education training to professionals in a variety of clinical contexts [16–18]. Specifically, the use of role-plays with SPs in training can increase clinicians’ confidence [19] and performance [20,21] in conducting informed consent discussions for clinical procedures. More recently, researchers have begun to employ role-plays in training programs for staff conducting informed consent for research [22–24].

Training clinical research staff in communicating informed consent may have additional benefits beyond improving the consent process. For example, communication training has been identified as one possible method to help increase enrollment of underrepresented populations in clinical trials [6]. Including community members, particularly those from underrepresented populations, in the design and development of training curricula may also help increase diversity among research participants [24], a continuing challenge for translational science [25]. Thus, including community members, as opposed to trained actors or staff members, as SPs for role-play exercises may have additional benefits.

This paper describes the development and evaluation of a community-engaged informed consent training program for clinical research staff. Our aims are to (1) assess the reach and self-reported effectiveness of the informed consent trainings and (2) describe the impact of including community stakeholders in trainings developed for clinical research professionals.

### Program Development

Tufts Clinical and Translational Science Institute (CTSI) has been conducting regular trainings for clinical research coordinators and other research staff on the practical skills of communicating informed consent since 2019. The trainings create a context where clinical research coordinators get to role-play the informed consent process with SPs who are community members who had been research participants themselves. The goal of these trainings is twofold: (1) to bridge the gap between knowledge of the history and rules around informed consent and the practical interpersonal skills needed and (2) to include in the training process members of the community who add value from their experiences as former research participants.

The genesis of the informed consent trainings stemmed from challenges identified by both research coordinators at Tufts Medical Center and the Tufts CTSI Steering Committee. The Steering Committee identified enrollment and retention of research participants as a consistent challenge, and coordinators expressed a need for more practice in informed consent. In response, the Community and Stakeholder Engagement, Regulatory Affairs, and Professional Education teams from the Tufts CTSI created an ad hoc group to develop a training to meet this need. From the beginning, the team decided that it was critical to include community stakeholders as part of the training.

Members of the Tufts CTSI Stakeholder Expert Panel (SEP) fully participated in the development and implementation of the informed consent training program. The Tufts CTSI SEP, launched in 2018, now includes a diverse group of 30 community members who engage in a variety of activities across Tufts CTSI, including advising researchers, reviewing grant applications and student thesis proposals, providing feedback on research tool development, and co-developing interventions and trainings. One of these SEP members also had previous experience as a SP for medical education and joined the planning team. The informed consent training program launched in April 2019 and was conducted in person with learners from Tufts Medical Center. Three SEP members were initially invited to be serve as SPs based on their experience as research participants and patient advocates. In August 2020, the trainings moved to a synchronous online platform. As the program grew, more SEP members were recruited to diversify the skills and life experiences of SPs including bilingual speakers. No additional requirements were imposed. Starting with 2021, the format of the training was redesigned to take advantage of the Tufts CTSI’s newly upgraded learning management platform, I LEARN, and to address identified areas for improvement. Specifically, SEP members noted that some of the learners were unprepared to participate in the simulated consent activity. SEP members were compensated for their time, including planning, participation in the sessions as SPs, and debriefing.

We prepare SEP members to participate as SPs through a one-hour session that provides an overview of the role-play exercises, introduces techniques for obtaining informed consent, offers examples of informed consent scenarios that they may simulate, and provides guidance for giving feedback (see Supplementary Materials). Unlike other programs that use standardized patients, the “simulated patients” employed by this training program are encouraged to vary the informed consent scenarios that they role-play with learners. The goal of these simulation exercises is to expose learners to different perspectives and experiences that more closely resemble potential real-life encounters (e.g., including categorical and specific rejections, hostility, patients wanting to skip critical parts of the consent form).

The redesigned course is offered in a blended synchronous/asynchronous format which draws on Kolb’s Experiential Learning Theory to facilitate learners through a cycle of experiencing, reflecting, thinking, and acting [26]. The asynchronous component includes a short, interactive multimedia tutorial covering didactic content on informed consent conversations (see Supplementary materials for examples) as well as the consent form to be used for the role-play. The design of the tutorial is informed by the principles of multimedia learning theory [27]. Participants are also required to upload proof they have completed basic human subjects research training, such as the Collaborative Institutional Training Initiative Biomedical Foundations or Social-Behavioral Education course. They receive the Zoom link for the synchronous session only after they have completed the tutorial and accessed the consent form document.

The one-and-a-half to two-hour synchronous sessions, held via Zoom, include a brief recap of the key takeaways from the pre-work tutorial and time for clarifying questions. Participants then move to break out rooms where they practice informed consent via role-playing with simulated research participants. The synchronous online format allowed for small group breakout rooms, removing the difficulty of finding separate spaces for multiple simultaneous activities. Virtual sessions addressed the transportation barriers of the CTSI’s geographically dispersed constituents.

In 2021, we differentiated the training into two versions: one covers the fundamentals of informed consent (Obtaining Informed Consent: A Practical Approach), and a separate “special topics” version focuses on specific informed consent scenarios, such as working with non-English speaking participants and interpreters, minors, and individuals with (temporarily) diminished capacity. The pre-work tutorials were edited to include additional didactic content relevant to the specific special topic, in addition to content from the fundamentals course (see Supplementary materials). The courses are offered in an alternating schedule so that there is at least one fundamentals and one special topics course offered per calendar year. Approximately one week after the training, the curriculum team invites the facilitators and SPs to a debrief meeting. The goals of these debrief meetings are to review participant feedback, identify strengths and challenges, and discuss strategies to improve the program. The debrief meetings are not focused on evaluating the performance of the SPs.

The move to a fully online training program also allowed us to expand the potential reach. We promoted the trainings locally through a combination of newsletters, announcements at local town hall meetings for the research community, and direct email from our research administration office. In addition, we promoted the trainings to the members of our clinical research network and the other regional CTSAs through direct email, newsletters, and events calendar announcements.

### Structure of the Role-Plays

The exact structure of the role-play breakouts has been the subject of continuous improvement based on feedback from participants, SPs, and facilitators. Each small group includes a staff facilitator, one or two SEP members, and three to five participants. Utilizing Zoom breakout rooms, each participant practices reviewing all or part of the consent form with the SEP members role-playing the SP. The SPs adopt different attitudes and perspectives on the study for each participant to explore varied dynamics in each session. Each role-play lasts between five and 10 minutes, depending on the number of participants in the group. After each participant has had a chance to role-play the consent conversation, the facilitator invites the SP to give 2–3 key takeaways of feedback, based on guidance provided during their orientation session (See Supplementary Materials for rubric). After all participants have completed the role-play, there is a group debrief session where the SP, facilitator, and other participants have an opportunity to provide additional feedback for all learners. The facilitator also has a rubric to guide their feedback (see Supplementary materials). Typically, the lead faculty (RS) spends some time observing in each breakout group. After the groups have finished, participants, SPs, and facilitators return to the main Zoom room and RS leads a general debrief.

### Program Participation and Evaluation

This paper covers the four trainings held between February 2021 and 2022 (see Table 1). Of those, two were offerings of the fundamentals course and two were special topics. Initially, we offered two sessions for each course in 2021 to make up for perceived demand due to a lack of trainings held in 2020, then switched to one session per course starting in September 2021. To assess program reach, we tracked the institutional affiliation for all attendees at the time of registration. While the majority of participants were from Tufts Medical Center or Tufts University (*N* = 41), across all six sessions we trained 76 unique individuals representing 11 different institutions, with seven individuals attending two or more courses, demonstrating our ability to reach participants both within and without our network. In order to maximize our reach for these voluntary opportunities, limited data were collected during registration; thus, a complete demographic profile of learners is not available. Promotion strategies for Tufts CTSI professional education programs vary according to the specific learner groups being targeted and the different capacity limitations set by the interactive components of some programs, so a direct comparison to the reach of other programs is not possible.

**Table 1.** Training topics, dates, and participation

Course title	Session date(s)	Total completing pre-work	Total attending live session
Obtaining informed consent: A practical approach	February 1, 2021	13	13
	February 4, 2021	15	14
Including non-English speakers in research^i^	June 8, 2021	15	11
	June 10, 2021	20	15
Obtaining informed consent: A practical approach	September 22, 2021	25	18
Obtaining informed consent with special populations^ii^	February 3, 2022	19	13
^i^Languages used were Cantonese, Mandarin, and Spanish. ^ii^Special populations targeted were minors and adults with temporarily diminished capacity.			

### Participant Evaluations

To assess self-reported effectiveness, we posted a link and projected a QR code linked to an anonymous evaluation questionnaire towards the end of every session. Participants were asked to complete the evaluation before leaving the Zoom, and a follow-up email was sent to all attendees the day after the session reminding them to complete the evaluation if they had not already done so.

The questionnaire for all four trainings consisted of two blocks of Likert-type questions. Block one asked participants to indicate the extent to which they agreed that the training had improved their abilities in the specified learning objectives (see Table 2). Block two asked the participants to rate their level of agreement on items related to the structure and format of the course (see Table 3). The learning objectives for both offerings of the fundamentals course were the same, while the two special topics courses had learning objectives tailored to the content. The questions related to format and structure were consistent across all four offerings. In addition, the questionnaires included optional open-ended questions asking participants to indicate the most valuable part of the course, suggestions for improvement, and to indicate one thing they will do in their practice as a result of taking the training.

**Table 2.** Participants’ ratings on course learning objectives

Obtaining informed consent: A practical approach, February 2021 & September 2021 (*N* = 34)					
Taking this course improved my ability to:	Strongly agree	Agree	Neither agree nor disagree	Disagree	Strongly disagree
List the essential elements of informed consent.	53%	41%			6%
Demonstrate (using plain language) explaining the informed consent process, in a virtual setting.	56%	35%	3%		6%
Identify 2-3 key considerations for obtaining informed consent in a remote or virtual setting.	59%	35%		3%	3%
Demonstrate multiple techniques to verify participants’ comprehension.	53%	35%	6%	6%	

Including Non-English Speakers in Research (*N* = 24)					
Taking this course improved my ability to:	Strongly agree	Agree	Neither agree nor disagree	Disagree	Strongly disagree
Identify when a participant needs an interpreter in order to obtain informed consent.	54%	25%	17%	4%	
Demonstrate the correct procedure for using an interpreter to obtain informed consent.	71%	25%		4%	
Demonstrate 3 communication techniques to use while obtaining informed consent with an interpreter.	75%	21%	4%		

Obtaining Informed Consent with Special Populations (*N* = 6)					
Taking this course improved my ability to:	Strongly agree	Agree	Neither agree nor disagree	Disagree	Strongly disagree
Demonstrate (using plain language) explaining the informed consent process, in a virtual setting.	33%	67%			
Demonstrate professional judgment on whether a potential participant has the capacity to, and is providing, willing and uncoerced consent.	17%	83%			
Distinguish between assent and consent to participate in research.	50%	50%			
Distinguish between competency and capacity with respect to informed consent conversations.	33%	50%	17%		

**Table 3.** Participants’ ratings on course format and structure

Obtaining informed consent: A practical approach, February 2021 & September 2021 (*N* = 35)					
	Strongly agree	Agree	Neither agree nor disagree	Disagree	Strongly disagree
The course was well organized.	49%	46%		3%	3%
The course was interactive and engaging.	69%	26%		3%	3%
The course stimulated new ideas/skills for my own research/practice	59%	32%	6%		3%
The material covered was relevant to my research, practice, or work.	54%	37%	3%	3%	3%
The blended synchronous/asynchronous format met my learning needs.	51%	40%	3%	3%	3%
The role-play activity in the live session was well organized and effective.	50%	33%		8%	8%
I would recommend this course to others.	54%	40%		3%	3%
Overall, I am satisfied with this course.	47%	47%		3%	3%

Including Non-English Speakers in Research (*N* = 24)					
	Strongly agree	Agree	Neither agree nor disagree	Disagree	Strongly disagree
The course was well organized.	75%	17%	4%	4%	
The course was interactive and engaging.	71%	25%	4%		
The course stimulated new ideas/skills for my own research/practice	79%	17%	4%		
The material covered was relevant to my research, practice, or work.	63%	29%	4%	4%	
The blended synchronous/asynchronous format met my learning needs.	71%	21%		8%	
The role-play activity in the live session was well organized and effective.	71%	21%	4%	4%	
I would recommend this course to others.	75%	17%		8%	
Overall, I am satisfied with this course.	71%	17%	4%	8%	

Obtaining Informed Consent with Special Populations (*N* = 6)					
	Strongly agree	Agree	Neither agree nor disagree	Disagree	Strongly disagree
The course was well organized.		83%	17%		
The course was interactive and engaging.	33%	67%			
The course stimulated new ideas/skills for my own research/practice	33%	67%			
The material covered was relevant to my research, practice, or work.	67%	33%			
The blended synchronous/asynchronous format met my learning needs.		100%			
The role-play activity in the live session was well organized and effective.		83%	17%		
I would recommend this course to others.	67%	33%			
Overall, I am satisfied with this course.	17%	67%	17%		

Overall, the response rate on the evaluations averaged 72.5 percent across all four trainings. Participants’ responses were overwhelmingly positive and indicated their agreement that the course was well-structured and improved their skills. In the open-ended responses, participants most frequently mentioned the role-play activity and interacting with the SPs, along with the opportunity to get feedback from the SPs and their peers, as the most valuable parts of the course. For example:

“The role-play was incredibly helpful; difficult but necessary. It was a struggle to explain the concepts of a biorepository and confidentiality, etc. It was good to see where my weaknesses were so I can work on those.”

“My simulated patient really challenged me and made me think a lot about how I introduce myself and approach a consent conversation.”

“[sic] Getting feedback as I have been consenting participants for years and it is easy to get into questionable habits.”

Other valuable aspects mentioned less frequently were the opportunity to practice, listening to and observing others, and the pre-work tutorial.

### Focus Group with Simulated Patients

All nine people who had been SPs were invited to take part in a focus group discussion. Six of them participated in a 60-minute focus group held over Zoom (See Table 4 for participant characteristics). The semi-structured interview guide explored their motivations for volunteering to be a SP, the challenges they experienced in participating, the perceived impact the training had on the learners, and the value of involving community members like themselves. The focus group discussion was recorded, transcribed, and de-identified to protect the confidentiality of the SEP participants. The transcript was analyzed thematically by two authors (NW, KM) to address our second aim of describing the impact of community member engagement on the trainings. All research protocols and materials were approved by the Tufts Health Sciences Institutional Review Board.

**Table 4.** Demographic characteristics of focus group participants (*N* = 6)

Age	
18–29	1
46–59	1
60+	4
Sex	
Female	4
Male	2
Race & Ethnicity	
White, non-Hispanic/Latino	4
White Hispanic/Latino	1
Black, non-Hispanic/Latino	1
Employment Status	
Retired	4
Full-time student	1
Unemployed	1
Current/Past Areas of Employment*	
Healthcare/medicine	4
Social services	2
Education	1
Other	2
Length of Membership in Tufts CTSI Stakeholder Expert Panel	
Less than 6 months	1
2–3 years	3
4+ years	2

*Participants could select more than one choice

Most SEP members involved in these trainings had been research participants themselves, which gave them unique insight into clinical trial enrollment and retention. Focus group participants described negative experiences with research that shaped their overall view of the research enterprise and highlighted the need to improve the way clinical research staff are taught to value patient perspectives and interact with participants who vary in age, education, literacy, language, and lived experience. Negative experiences included lack of empathy towards patient fears and concerns, maltreatment of or hostility towards patients, and failing to communicate with research participants “on their level.” These behaviors undermined participants’ ability to make informed decisions about research participation, leading some to disregard the informed consent process altogether. One participant reported:

“I’m not the most educated person… [A lot of trainees] ended up talking over my head so I couldnʼt understand really what was going on and it made me frustrated… I do a bunch of online surveys and that’s one of the reasons why I skip over the consent forms cause they’re all technobabble.”

Better understanding “what happens behind the curtain” of scientific research and improving the way human subjects research is conducted were described as some of the primary motivators for SEP members to join informed consent trainings. For some, this training allowed them to see the complexities of informed consent conversations from a different perspective – a chance rarely offered to community members. For others, serving as a SP allowed them to contribute to training the next generation of researchers to “do better” and to give voice to patients like themselves who make clinical research possible. As one participant put it:

“[My passion is to help] any kind of health professional to do better… with patients and families and everything like that. Having been a social worker and having been a cancer patient… my goal has always been to help them do better and to advocate for people.”

Overall, SPs viewed the informed consent trainings as an opportunity for clinical research staff to build necessary skills and confidence to talk through the risks, benefits, and logistics of taking part in clinical research in a way that addresses individual patients’ hopes and fears. Focus group participants emphasized that research staff should not only be knowledgeable about the research subject but also be able to communicate with empathy, transparency, and simplicity to enable individuals to make informed decisions about their own health and safety. One participant shared:

“It’s an opportunity to help them [coordinators] feel comfortable in their own skin and to give them enough grounding so that they are not timid… I get it, that they are young and relatively inexperienced, but this is a hard tool that they can use to be comfortable and also to learn that it’s okay to say, “I don’t know”… This is complicated stuff, and it’s a lot that weʼre asking of the person in front of us, and we owe it to them, the patient – the participant – to be completely transparent.”

Focus group participants felt that their lived experience strengthened informed consent trainings. By using community members like themselves, clinical research staff could practice informed consent conversations and receive feedback from “real people” who have been on the other side of those conversations before (i.e., past research participants). By bringing past experiences – positive and negative – into the training with them, SPs were able to give clinical research staff authentic feedback that would enhance their ability to adapt to the diversity existing in the “real world.” For example:

“We’re presented as stakeholders and community members so we’re closer to the real people they’re going to be trying to get consent from.”

“The value of bringing a diverse community in is that you’re getting very different experiential examples in front of these coordinators. So you’re getting very different levels of sophistication, education, experience on the patient’s part.”

Focus group participants felt that informed consent trainings using role-plays with past research participants are vital part of training clinical research staff, as it gives them an opportunity to practice these difficult-to-master skills and receive broad and immediate feedback. By creating a space where research coordinators can hone their skills and make mistakes, focus group participants hoped to reduce the risk that clinical research will lead to bad experiences.

“We don’t want them to be in a sink or swim situation. We want to give them the tools. So, it would seem to me this [training] should precede anybody ever getting in front of a live patient on a live project.”

## Conclusion

This report describes the process of developing a new training program for clinical research staff charged with enrolling patients in clinical trials. Tufts CTSI builds on existing education and training through a combination of experiential learning [26] and multimedia instruction [27] to fill a critical gap in professional development of the clinical research workforce. The goal of this effort is to increase participant understanding of research, communicate respect and enhance trust, and ultimately to improve our effectiveness in recruitment of a diverse group of research participants. By embedding community members in the trainings, clinical research coordinators hear diverse perspectives, experience a range of patient responses, and learn from the lived experience of the communities that research serves. Feedback from individuals who participated in the trainings as either learners or SPs was overwhelmingly positive and demonstrated the value and impact of this unique model for improving informed consent conversations. Clinical research staff consistently rated the training highly and reported that they would recommend the training to others. Open-ended feedback also showed that the role-plays and inclusion of community members as SPs were invaluable features of the training.

The training program described here positively impacted not only the clinical research staff who joined the training, but the SPs as well. Notably, SPs perceptions of the clinical research enterprise improved through their inclusion on the project. Participating in the training gave SPs an opportunity not often available to community members: to have a hand in improving the way clinical research is conducted. SPs reported a range of past research experiences and identities that gave them a unique perspective on how best to conduct informed consent conversations. By utilizing community members as trainers, we shifted traditional power dynamics and foregrounded community voices and patient perspectives. Including community members in the development, conduct, and assessment of the training is another way that Tufts CTSI implements the concept of broadly engaged team science [28] that respects and values community stakeholders.

There are limitations to consider with this report. This paper describes the development and evaluation of a pre-existing, voluntary training program promoted across our partner network, rather than a systematic educational intervention targeted at a prespecified group of research staff. Therefore, this precluded the use of a control group, formal assessments, and collection of detailed participant demographics. Observing learners’ actual informed consent conversations could have provided valuable data on the effects of this training; however, the logistical and ethical considerations of observing real patient-staff interactions made this infeasible. Further research will explore the relationship between the process described here and the outcomes of improved communication and recruitment. In addition, although six of the nine SPs chose to participate in the focus group discussion, the small number of participants prevents the disaggregation of data to examine the role of cultural, racial, ethnic, and gender identities.

Based on our experience and assessment of the Tufts CTSI community-engaged informed consent trainings, we recommend that all clinical research personnel who are involved in obtaining informed consent receive specific, experientially grounded training to develop the interpersonal skills required to communicate the information necessary for potential participants to make an informed decision. We further suggest that these trainings utilize community members and past research participants as SPs to improve the authenticity of informed consent role-plays and work towards dismantling harmful power dynamics. In the future, we plan to expand the training’s reach and impact through collaboration with other Clinical and Translational Science Award programs and inclusion of additional community voices.

## Supplementary material

For supplementary material accompanying this paper visit https://doi.org/10.1017/cts.2023.534. click here to view supplementary material
