# Supplementary material for: Community-engaged training in informed consent
Source: J Clin Transl Sci. 2023 Apr 20;7(1):e108. doi: 10.1017/cts.2023.534 (PMC10260332; doi:10.1017/cts.2023.534)
Supplement: Supplementary file 1 [file S2059866123005344sup001.docx]

**Community Engaged Training in Informed Consent Supplementary Materials**

**Sample Screenshots from Pre-work Tutorials**

**
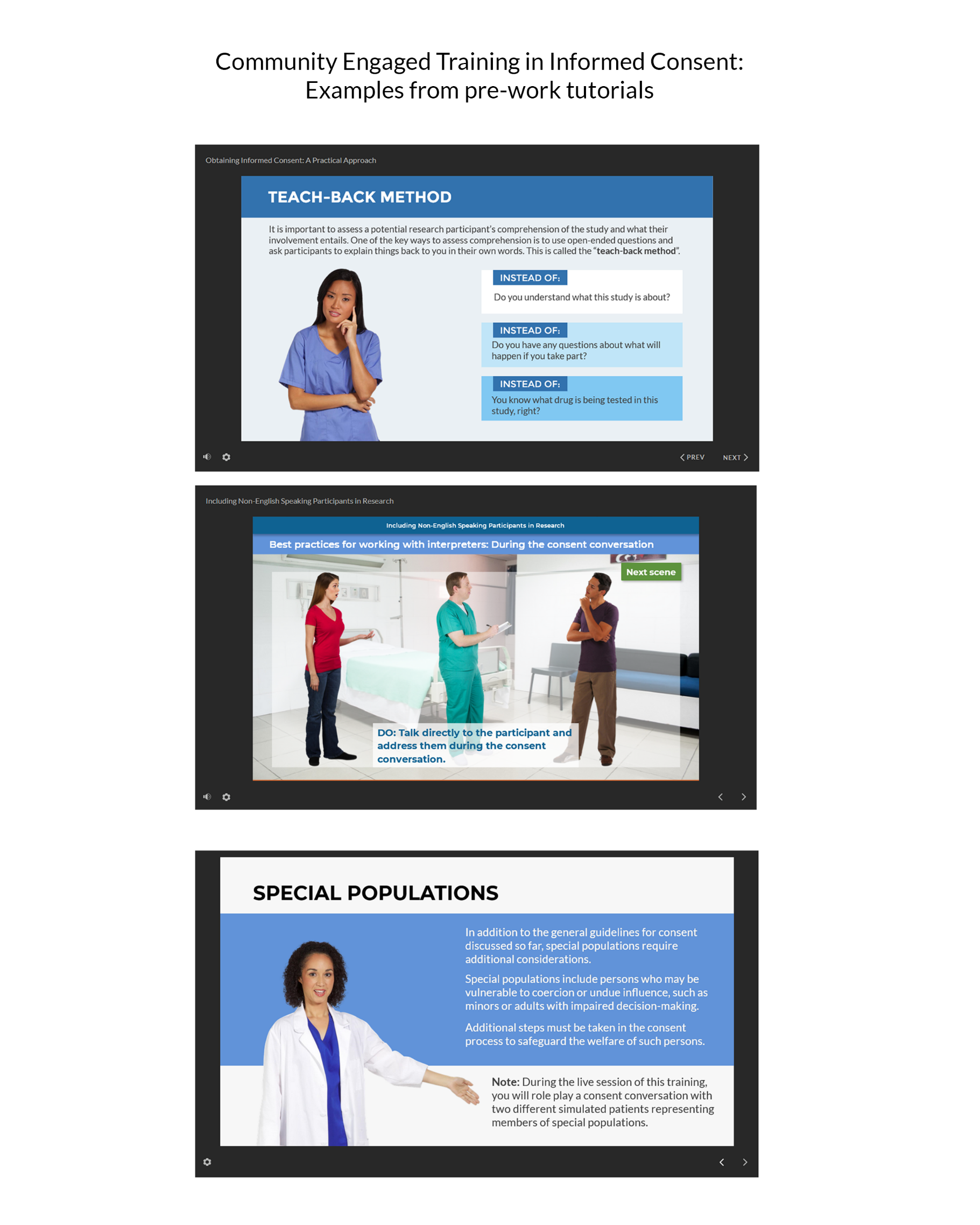
Feedback Rubrics**

**Feedback Guidance for Simulated Patients**

After each role-play, note your observations to the following questions:

- What did you find effective?
- Did you hear anything that others might want to incorporate into their own consent process?
- What would you suggest they do differently?
- Did the participant use teach back and/or comprehension checks at strategic times?
- Was the participant using plain language?

Structure your feedback using the “sandwich” technique: Start with a positive observation, include critiques in the middle, and end with another positive observation.

**Feedback Guidance for Facilitators**

During each role play, observe and take notes. Participants should:

- Introduce themselves
- Identify the person they are approaching in an open-ended way
- Clearly state what they are there for
- Answer questions correctly or offer to get more information if they can't answer a participant question
- Create a comfortable but professional interaction
- Demonstrate understanding of the protocol
- Use teach-back technique to check for comprehension
- Clarify the difference between the patient’s usual care and the research procedures
